# Corona- and Paramyxoviruses in Bats from Brazil: A Matter of Concern?

**DOI:** 10.3390/ani14010088

**Published:** 2023-12-26

**Authors:** Matheus Nunes Weber, Mariana Soares da Silva

**Affiliations:** Laboratório de Microbiologia Molecular, Universidade FEEVALE, Novo Hamburgo 93525-075, RS, Brazil; marianasilva2@feevale.br

**Keywords:** coronavirus, PMV, chiropterans, eco-vigilance, zoonosis

## Abstract

**Simple Summary:**

Because Brazil presents continental dimensions containing a rich biodiversity, this review aims to map the presence of coronavirus (CoV) and paramyxovirus (PMV) genetics related to human-relevant pathogens in bats. The CoVs and PMVs reported in Brazilian bats were genetically closely related to some human pathogens. The majority of works assayed phyllostomid, molossid and vespertilionid bats and found a majority of alpha-CoVs and few reports of beta-CoVs grouped in the *Merbecovirus* and *Embecovirus* subgenera, where MERS-CoV and HCoV OC43 are classified, respectively. The PMVs reported in Brazilian bats were classified in the Jeilongvirus and Morbillivirus genera. Despite the growing number of studies on CoVs and PMVs in bats in Brazil, when we compare the number of reports to the number of bat species found in Brazil, the representativeness of the viruses found and the available genomes, it can be perceived that there may be gaps in the knowledge. Therefore, it is necessary to pay attention and give relative importance and encouragement to future studies on bats, especially in relation to viruses with significant zoonotic potential, such as CoVs and PMVs.

**Abstract:**

*Chiroptera* are one of the most diverse mammal orders. They are considered reservoirs of main human pathogens, where coronaviruses (CoVs) and paramyxoviruses (PMVs) may be highlighted. Moreover, the growing number of publications on CoVs and PMVs in wildlife reinforces the scientific community’s interest in eco-vigilance, especially because of the emergence of important human pathogens such as the SARS-CoV-2 and Nipha viruses. Considering that Brazil presents continental dimensions, is biologically rich containing one of the most diverse continental biotas and presents a rich biodiversity of animals classified in the order *Chiroptera*, the mapping of CoV and PMV genetics related to human pathogens is important and the aim of the present work. CoVs can be classified into four genera: *Alphacoronavirus*, *Betacoronavirus*, *Deltacoronavirus* and *Gammacoronavirus*. Delta- and gammacoronaviruses infect mainly birds, while alpha- and betacoronaviruses contain important animal and human pathogens. Almost 60% of alpha- and betacoronaviruses are related to bats, which are considered natural hosts of these viral genera members. The studies on CoV presence in bats from Brazil have mainly assayed phyllostomid, molossid and vespertilionid bats in the South, Southeast and North territories. Despite Brazil not hosting rhinophilid or pteropodid bats, which are natural reservoirs of SARS-related CoVs and henipaviruses, respectively, CoVs and PMVs reported in Brazilian bats are genetically closely related to some human pathogens. Most works performed with Brazilian bats reported alpha-CoVs that were closely related to other bat-CoVs, despite a few reports of beta-CoVs grouped in the *Merbecovirus* and *Embecovirus* subgenera. The family Paramyxoviridae includes four subfamilies (*Avulavirinae*, *Metaparamyxovirinae*, *Orthoparamyxovirinae* and *Rubulavirinae*), and bats are significant drivers of PMV cross-species viral transmission. Additionally, the studies that have evaluated PMV presence in Brazilian bats have mainly found sequences classified in the *Jeilongvirus* and *Morbillivirus* genera that belong to the *Orthoparamyxovirinae* subfamily. Despite the increasing amount of research on Brazilian bats, studies analyzing these samples are still scarce. When surveying the representativeness of the CoVs and PMVs found and the available genomic sequences, it can be perceived that there may be gaps in the knowledge. The continuous monitoring of viral sequences that are closely related to human pathogens may be helpful in mapping and predicting future hotspots in the emergence of zoonotic agents.

## 1. Introduction

Most emerging infectious diseases are zoonotic, with ~70% originating in wildlife [1]. Cross-species transmission and eco-vigilance are challenging and the least-studied aspects of disease ecology, yet cross-species transmission is the defining process in zoonotic spillover and disease emergence [2,3,4]. Rodents and chiropters are the two most diverse mammal orders, with over 2000 and 1100 species, respectively [5,6]. Chiropters also share several characteristics that have been hypothesized to affect reservoir potential, such as hosting a taxonomic order that is evolutionarily ancient and diverse and includes many species with peri-domestic habits and species that commonly express torpor [6]. Bats are even more likely to be speculated as unique in their potential to harbor zoonotic viruses because of characteristics such as flight, which allows movement and dispersal over long distances in some species [6]. Moreover, many bat species are gregarious, some living in dense aggregations and cohabiting roosting sites with diverse assemblages of multiple bat species [6,7,8]. Additionally, their unique immune system that limits self-damaging inflammatory responses makes bats more likely to host zoonotic viruses in particular and/or transmit them to humans [9,10,11].

Chiropterans are considered reservoirs of main human pathogens, including the Ebola and Marburg filoviruses and Nipah and Hendra paramyxoviruses, as well as bridge hosts of SARS coronavirus (SARS-CoV), SARS-CoV-2 and Middle East respiratory syndrome coronavirus (MERS-CoV) [5,6,12,13]. Additionally, bats appear to be ancient natural reservoirs of several viral families, including hepaciviruses, pegiviruses and coronaviruses [14,15,16]. Bats are also considered a main ancient natural reservoir of paramyxoviruses and influenza A viruses [16,17]. Additionally, their presence in urban, wild and rural environments facilitates viral transmission to intermediate hosts, which has been crucial in understanding the important zoonotic viruses’ spillover mechanism to humans, which includes CoVs and PMVs [2,3].

Brazil is a large country with continental dimensions (~8512,000 km^2^) containing predominantly tropical areas with extensive forested areas in the Amazon region as well as remains of a rainforest on the eastern, southeastern and southern coasts. There is a wide variability in the climates around the country, which provides an auspicious environment for diverse flora and fauna, highlighting the Amazon (north region), Caatinga (northwest), Pantanal (central west) and Pampa (south) biomes. Brazil presents about 751 listed wild mammal species that are distributed in 249 genera and 11 orders, among which 182 are classified in the order *Chiroptera* [18]. The variety of this important mammal order that potentially harbors important human pathogens increases the necessity of eco-vigilance and monitoring virus genetics that are closely related to human pathogens in Brazil. In this context, this review addresses relevant aspects of the CoVs and PMVs in chiropterans with an emphasis on Brazilian territory reports and provides an update on the information available in the scientific literature in order to map the presence of virus genetics related to human-relevant pathogens.

## 2. Coronaviruses

Bats have been implicated as major reservoirs of coronaviruses in nature and a source of zoonotic transfer to humans [19]. In fact, almost 60% of alpha- and betacoronaviruses are related to bats, which are considered natural hosts of the members of these viral genera [20,21]. Coronaviruses are single-stranded positive-sense RNA viruses with genomes of 16 to 31 kb. Members of the family *Coronaviridae* (realm *Riboviria*, kingdom *Orthornavirae*, phylum *Pisuviricota*, class *Pisoniviricetes*, order *Nidovirales* and suborder *Cornidovirineae*) can infect a wide range of animals and are generally associated with respiratory and/or digestive illness [22,23,24]. CoVs are divided into four genera, of which only members of the *Alphacoronavirus* (α-CoV) and *Betacoronavirus* (β-CoV) genera are known to infect humans [22,23]. The *Deltacoronavirus* and *Gammacoronavirus* genera are more likely to infect birds [22,23,24]. Some coronaviruses are associated with the endemic common cold in humans (i.e., HCoV-229E and HcoV-NL63—α-CoVs; HcoV-HKU1 and HcoV-OC43—β-CoVs) [22,23,25,26,27,28]. Phylogenetic analyses indicate that bats most likely represent the ultimate animal reservoirs from which the HcoV-NL63 and HcoV-229E α-CoVs emerged [27,28,29].

SARS-CoV, SARS-CoV-2 and MERS-CoV β-CoVs are strongly associated with severe respiratory diseases in humans and present their origin in bat bridge hosts [12,30,31]. In fact, bats of the family Rhinolophidae are natural hosts to a suite of sarbecoviruses (subgenus within the *Betacoronavirus* genus wherein SARSr-CoV are classified) and considered to be at high risk for the emergence of novel β-CoVs that pose a high risk to human health [12,30,31]. These bats are not found in the Americas. They inhabit the Old World, including Africa, Australia, Asia, Europe and Oceania [32]. On the other hand, bats species in the family *Vespertilionidae* are widely considered to be the evolutionary source of MERS-CoV or its immediate ancestor [33,34] and are distributed worldwide [18].

### Brazilian Bat Coronaviruses

CoV surveillance studies were carried out in bats in Brazil accessing the Amazonian, Atlantic Forest and Cerrado biomes in different habitats including preserved wild areas, rural, peri-urban and urban environments [35,36,37,38,39,40,41,42,43]. These works are still incipient, assessing mainly phyllostomid, molossid and vespertilionid bats in the South [39,41,44,45], Southeast [35,36,38,40,41,42] and Northeast [37,41,44] territories using fecal, oral and tissue samples. The bat diets varied from omnivorous, frugivorous, nectarivorous and insectivorous to hematophagous [35,36,37,38,41,43,45]. Specifically, α-CoVs have already been detected in *Artibeus cinereus*, *Artibeus lituratus*, *Carolia brevicauda*, *Carolia perspicillata*, *Desmodus rotundus*, *Glossophaga soricine*, *Phyllostomuns discolor*, *Phyllostomus hastatus* and *Sturnira lilium* bat species from the Phyllostomid family; *Cynomops planirostris*, *Molossus currentium*, *Molossus molossus*, *Molossus rufus* and *Tadarida brasiliensis* bat species from the Molossidae family; and *Myotis nigricans* and *Myotis riparius* bat species from the Vespertilonidae family [35,36,37,38,39,40,41,42,43,44,45]. Β-CoV has been already described in *Desmodus rotundus* [36] and *Artibeus lituratus* [42] species from the Phyllostomidae bat family and *Eumops glaucinus* [42] species from the Mollossidae bat family.

**Figure 1:**
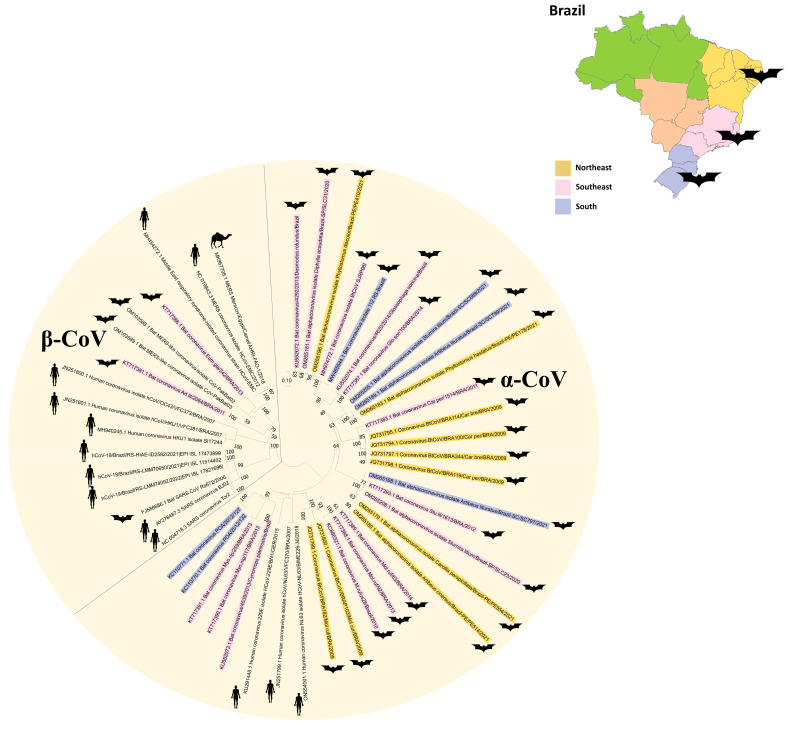
Coronavirus phylogenetic tree from the subfamily *Coronavirinae*, including representatives of two genera: α-CoV and β-CoV. Brazilian bat coronavirus sequences are colored according to the region: Northeast (yellow), Southeast (pink) and South (blue). The Maximum Likelihood phylogenetic analysis under the General Time Reversible for a proportion of invariable sites, applying 200 replicates, 1000 bootstraps.

As observed in Figure 1, most of the bat-CoV sequences found in Brazil belong to the α-CoV genus, and three sequences of the β-CoV genus were detected in *Desmodus rotundus* [36], *Artibeus lituratus* and *Eumops glaucinus* [42]. Out of these three sequences, the β-CoV reported in *E. glaucinus* belongs to Merbecovirus and is closely related to Bat MERS-like CoV (~88%) in the RNA-dependent RNA polymerase region. The *A. lituratus* CoV is also clustered in the Merbecovirus subgenus but with a ~69% nucleotide identity with MERS-CoV. The *D. rotundus* β-CoV belongs to the Embecovirus subgenus, presenting a 93.6% nucleotide identity with HCoV-OC43. Because of the sequence length, this sample was not included in the phylogenetic analysis. None of the Brazilian bat-CoVs were clustered in the Sarbecovirus subgenus. The majority of α-CoVs detected in Brazilian bats were more closely related only to other bat-CoVs and not to HCoVs [35,37,38,39,40,41,42,44,45], in contrast to an α-CoV detected in *Tadarida braziliensis* that was closely related to the Appalachian Ridge CoV strain 2 from which HCoV-NL63 probably evolved [46]. There are also non-published works with a few sequences deposited in the ZOVER database [47]. It is important to note that Figure 1 clearly shows the diversity of CoVs in the different Brazilian regions, because there are no defined specific CoV clusters in each region. This underscores the genetic variability of CoVs distributed across the country.

## 3. Paramyxoviruses

Paramyxoviruses are a diverse group of negative-sense, single-stranded RNA viruses of which several species cause significant mortality and morbidity. The family *Paramyxoviridae* (realm *Riboviria*, kingdom *Orthornavirae*, phylum *Negarnaviricota*, subphylum *Haploviricotina*, class *Monjiviricetes*, order *Mononegavirales* and family *Paramyxoviridae*) is comprised of four subfamilies: *Avulavirinae*, *Rubulavirinae*, *Metaparamyxovirinae* and *Orthoparamyxovirinae*. To date, the subfamily *Orthoparamyxovirinae* is comprised of eight genera: *Respirovirus*, *Aquaparamyxovirus*, *Ferlavirus*, *Henipavirus*, *Narmovirus*, *Jeilongvirus*, *Salemvirus* and *Morbillivirus* [48]. Important PMVs associated with disease in humans include *Rubulavirus*, which contains the mumps virus; *Respirovirus*, formerly known as human parainfluenza virus, a common cause of childhood respiratory disease; *Morbillivirus*, where the measles virus is grouped; and *Henipavirirus*, which contains pathogens causing fatal encephalitis in humans such as *Hendra virus* (HeV) and *Nipah virus* (NiV) [49,50].

Outbreaks of zoonotic and highly lethal orthoparamyxoviruses, such as Hendra and *Nipah virus*, have highlighted the importance of bat surveillance studies to prepare for the future emergence of yet unknown PMVs from wildlife reservoirs, as these animals have a wide range of species and are significant drivers of PMV cross-species viral transmission [16,51,52,53]. According to Drexler et al. [16], shifts in paramyxovirus host to other mammalian species are primarily from bats. Several PMVs have been involved in deadly zoonoses in humans in recent years, such as HeV and NiV [50,51,53], which have been sporadically acquired from pteropodid bats by humans, swine and horses. These outbreaks have attracted growing interest in zoonotic PMVs. Furthermore, recent works revealed many novel PMVs in wild mammals. Most sampling efforts have focused on bats because of their diversity and abundance and the significance of their role as hosts for other viral zoonoses [16,53].

Recently, the PMVs classified into the *Jeilongvirus* genus have emerged from an extensive range of small mammals, including bats [16,53,54,55,56]. This genus was discovered in 1977 in a kidney autoculture of a dead house mouse, *Mus musculus*, in Northern Queensland, Australia [57]. Some jeilongviruses such as *Beilong virus* (BeiV) and J paramyxovirus (JPV) have been hypothesized as having the potential for zoonotic spread to humans. Serological evidence suggests that JPV has previously spilled over into the human population, although only limited to Australia [57], while BeiV is capable of cross-contaminating human cell cultures from rodent cell cultures [58].

### Brazilian Bat Paramyxoviruses

Bat PMV surveillance performed in Brazil has been mainly performed in the Amazonian, Atlantic Forest and Cerrado biomes [16,45,54,55,56]. Jeilongvirus and the putative Jeilongvirus-related genus were the most common PMVs detected in Brazilian bats, which included *Corollia perspicillata* [16,54,56], *Corollia brevicauda* [16], *Desmodus rotundus* [16,55], *Glossophaga soricina* [16] and *Diameus youngi* [54,55] species from the *Phyllostomidae* family versus one report in a *Tadarida braziliensis* molossid bat [45]. The *Morbillivirus* genus was also detected in the *Phylostomidae* family, including *Desmodus rotundus* [16] and *Phyllostomus hastatus* [54], and also in the *Myotis riparius* chiropter bat [54].

The majority of sequences were classified as *Jeilongvirus* and the putative *Jeilongvirus-related* genus, instead of some Morbillivirus or morbillivirus-like sequences, and grouped with other bat and wild animal sequences. However, some morbilliviruses reported in *Desmodus rotundus* in Brazil were closely related to canine distemper virus [16]. *Morbillivirus* detection is always a main concern because some members of this PMV genus such as the measles virus, canine distemper virus and rinderpest virus cause severe disease in their natural hosts [53,54,59,60]. NiV-positive serology was also reported in phyllostomid bats (*Artibeus planirostris, Carollia perspicillata, Desmodus rotundus* and *Glossophaga* sp.) in Southeastern Brazil in the Cerrado biome [61].

Despite the absence of pteropodid bats in the Americas, which are known to be reservoirs of henipaviruses, the presence of henipa-like virus reservoirs in the Western hemisphere warrants further investigation. Moreover, the recent report of a novel henipa-like virus (putatively named peixe boy virus) in opossums (*Marmosa demerarae*) from a forest fragment area in Southeast Brazil reinforced this point [62]. In addition to the published works, there are still sequences deposited in the ZOVER database from research that has not yet been published.

## 4. Unlocking the Vast Potential for Viral Detection in Brazilian Bats

Despite hosting a wide variety of viruses, bats that have been surveyed seldom show signs of disease. Various hypotheses have been suggested to account for these asymptomatic infections. One theory suggests that bats, being the only flying mammals, generate substantial quantities of reactive oxygen species (ROS). In response, they have adjusted their genes to mitigate oxidative stress [63], potentially leading to decreased viral replication and pathogenicity [64]. The other hypothesis is related to a modified innate immune response and the constitutive expression of bat interferon subtypes that likely restrict disease while allowing for persistent low-level viral infections [63,65]. Cross-species transmission from bats is another important topic regarding emerging viruses’ transmission [16,52,53,66]. Effective transmission to humans frequently involves an intermediary or amplifying host. Optimal intermediary hosts are those species that engage with both the reservoir hosts (bats) and humans. Animals in intimate proximity to humans, such as domesticated species like pigs, horses and dromedary camels, play a significant role in the transmission of diseases from animals to humans [66]. SARS-CoV and SARS-CoV-2 are examples of the *Sarbecovirus* subgenus that have spread to humans, most likely via intermediate hosts [67,68].

**Figure 2:**
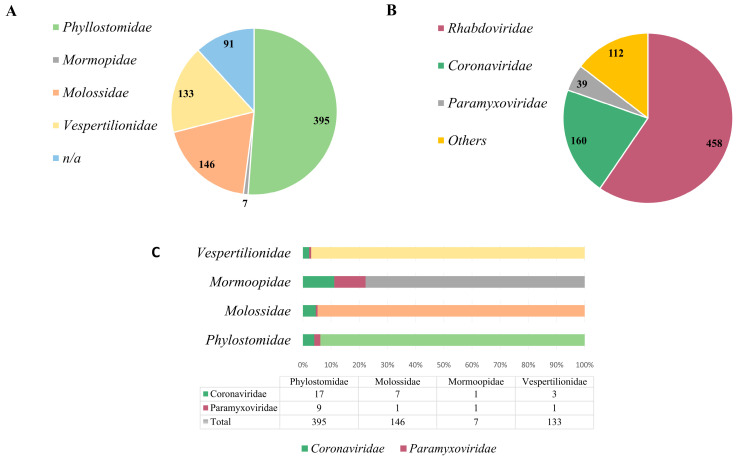
Brazilian bat species and bat-related virus data. (**A**) Represents each bat species within the families described in Brazil. (**B**) Represents the number of genome sequences deposited in the GenBank and ZOVER databases within the mentioned viral families. (**C**) The representativeness with which viral families were detected in each bat species.

Numerous research investigations have emphasized the heightened susceptibility of chiropterans to harboring zoonotic viruses and disseminating them to human populations [5,6,16]. Conversely, the study of other intermediary hosts and the identification of zoonotic viruses harbored by several mammalian species indicate that viruses engaged in transmission among diverse hosts eventually find their way into the human population [67,68,69], which is impactful in the extant fauna in Brazil. Research on viruses in bats is unevenly distributed across the world. Most studies are concentrated in Asia [70], Africa [71] and Europe [72]. South America, which is home to numerous bats, as observed in Brazil, receives relatively little attention from researchers. Because Brazil has continental dimensions and some of the most extensive and diverse fauna in the world, we highlight the currently limited and biased character of the research on the Brazilian bat, as observed in Figure 2A.

Currently, there are four bat families represented in the country: *Phylostomidae* (representing the majority, comprising 51% of the identified species), followed by *Molossidae* (9%), *Vespertilionidae* (17%) and *Mormoopidae* (1%), in addition to unidentified species, which comprise 12% [47] (Figure 2A). Phylostomids are abundant in tropical areas and have diverse eating habits, ranging from insectivorous, frugivorous animals to even blood-sucking bats, such as *Desmodus rotunduns*. Among the viral families already identified in bats in Brazil, three are more prominently represented: *Rhabdoviridae* (59%), *Coronaviridae* (21%) and *Paramyxoviridae* (5%) [47], which are significant viral families associated with diseases of importance in both animal and human health (Figure 2B). Other viral families comprise 15%. However, when we assess the representation of studies conducted on different bat species in relation to the identified viral families, it becomes apparent that Brazil lacks further research on these animals, and they potentially serve as reservoirs for a vast array of unidentified and uncharacterized viral agents. As observed in Figure 2C, in the *Phylostomidae* family which is represented by almost 400 bat species, PMVs or CoVs were detected in only 26 of them. The same pattern is repeated in the others, in which the *Molossidae* family is represented by more than 140 species of bats, as well as the *Vespertinolodae*, which has more than 130 cataloged species, and CoVs or PMVs have been described in less than 10 of these species until the present moment. From these data, we can probably characterize an underestimated detection rate. This presents a significant issue, given the vast expanse of the country, the diversity of its fauna and the zoonotic potential of many of these agents.

For most bat species, interactions with humans and other animals are limited to occasional occurrences such as through animal carers and in backyards and households. However, some bat species with a primarily frugivorous diet present indirect contact with other animal species which may favor spillover events. Moreover, some Molossidae bats are synanthropic species and inhabit houses and house attics, presenting close contact with humans. In contrast, hematophagous bats such as the common vampire bat (*Desmodus rotundus*), hairy-legged vampire bat (*Diphylla ecaudata*) and white-winged vampire bat (*Diaemus youngi*) from South and Central America have a unique blood-feeding diet that provides an opportunistic route for the transmission of viral agents such as rabies [48] but also other microbiota. In Brazil, the bats sampled in CoV and PMV studies have been hematophagous, insectivorous, frugivorous and nectivorous (Table 1 and Table 2), which reinforces the biodiversity available in the Brazilian territory. However, these different dietary habits provide multiple opportunities for contact between bats and other animals, providing cross-species transmission opportunities and making the eco-vigilance of virus genetics related to human and animal pathogens an important tool for mapping future potential pathogens.

**Figure 3:**
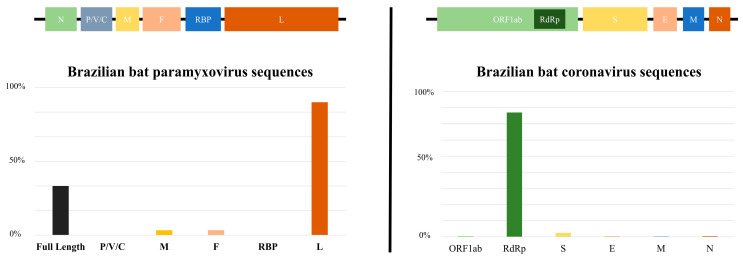
The bar chart depicts the number of sequences deposited in databases (*Paramyxoviridae* and *Coronaviridae* families) within each genomic region. P/V/C (polymerase-associated protein with alternative codons), M (matrix), F (fusion), RBP (receptor-binding protein), L (large), ORF1ab (nonstructural proteins), RdRp (RNA-dependent RNA polymerase), S (spike), E (envelop), M (membrane), N (nucleocapsid).

When comparing the quantity of genomic data on CoVs and PMVs in bats in Brazil, as published in the GenBank and ZOVER databases, it is evident that CoVs (160 sequences) exhibit a greater representation in comparison to PMVs (39 sequences) [47]. Furthermore, as can be observed in Figure 3, the sequences are concentrated in certain regions of the genome, and there is a scarcity of complete genomes, which poses challenges to genomic surveillance in the country. The limited scope of research on CoVs and PMVs in bats within Brazil raises concerns considering the paramount significance of these viruses due to their zoonotic potential, evolutionary adaptability and interspecies transmission capabilities. This knowledge gap not only poses a threat to wildlife but also has implications for domestic animals and human populations. Comprehensive studies are crucial to understand and monitor these viral dynamics, as they have the potential to impact public health and disrupt ecosystems and necessitate proactive measures to mitigate the risks associated with these infectious agents.

Brazilian bat genomic sequences of PMVs are mainly represented by large gene (L) and a few full-length genomes, and those of CoVs are basically comprised of RdRp (RNA-dependent RNA polymerase) sequences. It is important to mention that spike protein gene (S) sequences are scarce. When observing the limited number of these sequences, it is necessary to draw attention to the need for further studies and investigations in this genomic region. The primary factor influencing the host specificity of a coronavirus (CoV) is the trimeric spike (S) glycoprotein located on the surface. This glycoprotein can be subdivided into an N-terminal S1 subunit and a membrane-embedded C-terminal S2 region [73]. Exploring the diverse spike S1 portion of bat coronaviruses to detect viruses capable of binding to human receptors is crucial in assessing potential threats. Simultaneously, targeting evolutionarily conserved genes like the S2 region of the spike enables the development of therapeutics with broad efficacy against existing and potential future coronaviruses, offering a strategic approach to understanding, preparing for and potentially mitigating future disease outbreaks originating from bat sources amid global challenges such as habitat loss and uneven public health infrastructures [74].

## 5. Conclusions

Considering that Brazil is biologically rich, containing one of the most diverse continental biotas [18], a significant number of novel virus genetics related to human pathogens could be identified in the near future, particularly in the Amazon and Atlantic Forest biomes [4,35,38,39,42,45]. These works performed with Brazilian bats are still incipient, assessing mainly phyllostomid, molossid and vespertilionid bats in the South, Southeast and North territories [35,36,38,39,40,41,43,54,55,56]. Progress in molecular methods is necessary to enable not just the identification of the host species for coronaviruses but also the elucidation of the mechanisms through which bats either facilitate or impede the replication of these viruses [9]. The majority of works performed with Brazilian bats reported that α-CoVs are closely related to other bat-CoVs, despite a few reports of β-CoVs grouped in the *Merbecovirus* and *Embecovirus* subgenera (where MERS-CoV and HCoV-OC43 are classified, respectively). Rhinophilidae bats, which are the main natural hosts to a suite of sarbecoviruses (subgenus within the *Betacoronavirus* genus wherein SARSr-CoVs are classified), are not found in the Americas, and this fact explains the absence of reports of this CoV subgenus in Brazil [47].

Paramyxoviruses have a broad host range and geographic distribution, including human pathogens transmitted by bats such as Nipah and Hendra viruses. There is strong evidence of bats being in a close evolutionary and ecological relationship with several genera of mammalian PMVs [16]. The works that have evaluated PMV presence in Brazilian bats have mainly found sequences classified in the *Jeilongvirus* and *Morbillivirus* genera [16,45,53,54,55]. Morbillivirus detection is always a main concern because some members of this PMV genus such as measles virus, canine distemper virus and rinderpest virus cause severe disease in their natural hosts [49,50,59,60]. *Henipavirus* serology was also previously reported in phyllostomid bats in Brazil, despite the absence of pteropodidae bats in the Americas, which act as reservoirs of NiV. Moreover, the recent report of a novel henipa-like virus in *Marmosa demerarae* in Brazil [72] reinforces the need for the eco-vigilance of Brazilian wildlife. Using this review as a basis, we strongly suggest consistent genomic surveillance employing algorithms such as MetaBat, which facilitates the analysis of vast genomic data for the identification and classification of potential threats [75], which is essential for comprehensively monitoring and analyzing viral diversity in bat populations.

Bats constitute a significant yet inadequately understood reservoir of identified human pathogens. Despite the limited knowledge about bats, the viruses they harbor and the underlying molecular and ecological factors influencing viral spillover, advancements in tools for developing cutting-edge vaccines and antiviral technologies are progressing. These advancements enable researchers to respond to future outbreaks with unprecedented speed [52,72]. The growing number of publications on CoVs and PMVs in wildlife reinforces the scientific community’s interest in eco-vigilance, especially considering the emergence of important human pathogens such as SARS-CoV-2 and NiV. Because of the relative scarcity of studies on bats in Brazil, the continuous monitoring of viral sequences that are closely related to human pathogens may be helpful in mapping and predicting future hotspots in the emergence of zoonotic agents. As approximately 80% of human viral infections originate from animals (zoonotic diseases), it is crucial to conduct field surveys by monitoring these animal populations in their natural or adapted environments. Consequently, understanding the ecology of potential reservoir species and the primary pathways of transmission between different species is essential for effective preventive measures. It is important to highlight that the present work does not intend to villainize or promote any culling of bats, rodents or wildlife. Bats play important ecological roles in forest regeneration, acting in pollination and seed dispersion and in insect and pest control [75,76]. Thus, the conservation of these mammals is crucial in terms of One Health aspects. In fact, anthropization and forest fragmentation are the main villains in inducing zoonotic spillovers [70]. To conclude, in this review, we indicate that when comparing the bat species diversity data cataloged in Brazil with the viral ecology studies performed, the Brazilian data is probably underestimated. Therefore, it is presumable that Brazil has a much greater diversity of CoVs and PMVs than represented so far.

## Figures and Tables

**Table 1:** Bat species in which natural coronavirus infection has been detected in Brazil.

Genus	Host (Species/Family)	Primary Feeding Habit	Sample	Year	Region	Citation
β-CoV	*Desmodus rotundus*/Phyllostomidae	Hematophagous	Feces	2007	Southeast	[36]
α-CoV	*Carollia brevicauda*/Phyllostomidae	Frugivorous	Intestine	2009	Northeast	[37]
α-CoV	*Carollia perspicillata*/Phyllostomidae	Frugivorous	Intestine	2009	Northeast	[37]
α-CoV	*Molossus currentium*/Molossidae	Insectivorous	Intestine	2009	Northeast	[37]
α-CoV	*Molossus rufus*/Molossidae	Insectivorous	Intestine	2009	Northeast	[37]
α-CoV	*Molossus molossus*/Molossidae	Insectivorous	Intestine	2014	Southeast	[42]
α-CoV	*Molossus rufus*/Molossidae	Insectivorous	Intestine	2014	Southeast	[42]
α-CoV	*Molossus molossus*/Molossidae	Insectivorous	Feces	2012	South	[44]
α-CoV	*Tadarida brasiliensis*/Molossidae	Insectivorous	Feces	2012	South	[44]
α-CoV	*Cynomops planirostris*/Molossidae	Insectivorous	Feces	2014	Southeast	[35]
α-CoV	*Cynomops abrasus*/Molossidae	Insectivorous	Feces	2013	Southeast	[35]
α-CoV	*Desmodus rotundus*/Phyllostomidae	Hamatophagous	Feces	2013	Southeast	[35]
α-CoV	*Glossophaga soricina*/Phyllostomidae	Frugivorous	Feces	2014	Southeast	[35]
β-CoV	*Artibeus lituratus*/Phyllostomidae	Frugivorous	Intestine	2010–2012	Southeast	[42]
α-CoV	*Carollia perspicillata*/Phyllostomidae	Frugivorous	Intestine	2011–2012	Southeast	[42]
β-CoV	*Eumops glaucinus*/Molossidae	Insectivorous	Intestine	2013	Southeast	[42]
α-CoV	*Glossophaga soricina*/Phyllostomidae	Frugivorous	Intestine	2014	Southeast	[42]
α-CoV	*Molossus rufus*/Molossidae	Insectivorous	Intestine	2013–2014	Southeast	[42]
α-CoV	*Myotis nigricans*/Vespertilionidae	Insectivorous	Intestine	2013	Southeast	[42]
α-CoV	*Myotis riparius*/Vespertilionidae	Insectivorous	Intestine	2013	Southeast	[42]
α-CoV	*Sturnira lilium*/Phyllostomidae	Frugivorous	Intestine	2012	Southeast	[42]
α-CoV	*Phyllostomus discolor*/Phyllostomidae	Nectarivore	Intestine	2019	Southeast	[38]
α-CoV	*Tadarida brasiliensis*/Molossidae	Insectivorous	Oral	2014	South	[45]
α-CoV	*Desmodus rotundus*/Phyllostomidae	Hematophagous	Intestine	2019	South	[39]
α-CoV	*Artibeus cinereus*/Phyllostomidae	Frugivorous	Mix	2021	Northeast	[41]
α-CoV	*Artibeus lituratus*/Phyllostomidae	Frugivorous	Mix	2021	Northeast	[41]
α-CoV	*Carollia perspicillata*/Phyllostomidae	Frugivorous	Mix	2021	Northeast	[41]
α-CoV	*Phyllostomus discolor*/Phyllostomidae	Nectarivore	Mix	2021	Northeast	[41]
α-CoV	*Phyllostomus hastatus*/Phyllostomidae	Nectarivore	Mix	2021	Northeast	[41]
α-CoV	*Sturnira lilium*/Phyllostomidae	Frugivorous	Mix	2021	Northeast	[41]
α-CoV	*Artibeus lituratus*/Phyllostomidae	Frugivorous	Mix	2021	South	[41]
α-CoV	*Sturnira lilium*/Phyllostomidae	Frugivorous	Mix	2021	South	[41]
α-CoV	*Diphylla ecaudata*/Phyllostomidae	Hematophagous	Mix	2021	Southeast	[41]
α-CoV	*Sturnira lilium*/Phyllostomidae	Frugivorous	Mix	2021	Southeast	[41]

α-CoV—alphacoronavirus; β-CoV—betacoronavirus; Mix: oral and/or rectal and/or intestine.

**Table 2:** Bat species in which natural paramyxovirus infection has been detected in Brazil.

Genus	Host (Species/Family)	Primary Feeding Habit	Sample	Year	Region	Citation
*Jeilongvirus*-related	*Carollia perspicillata*/Phyllostomidae	Insectivorous	Tissue	2009	Northeast	[16]
*Jeilongvirus*-related	*Carollia brevicauda*/Phyllostomidae	Insectivorous	Tissue	2009	Northeast	[16]
*Jeilongvirus*-related	*Desmodus rotundus*/Phyllostomidae	Hematophagous	Tissue	2009	Northeast	[16]
*Morbillivirus*	*Desmodus rotundus*/Phyllostomidae	Hematophagous	Tissue	2009	Northeast	[16]
*Jeilongvirus*-related	*Glossophaga soricina*/Phyllostomidae	Frugivorous	Tissue	2009	Northeast	[16]
*Jeilongvirus*-related	*Tadarida brasiliensis*/Molossidae	Insectivorous	Oral Swab	2014	South	[45]
*Jeilongvirus*-related ^a^	*Carollia perspicillata*/Phyllostomidae	Insectivorous	Kidney	2013–2014	Southeast	[56]
*Jeilongvirus*-related ^a^	*Desmodus rotundus*/Phyllostomidae	Hematophagous	Kidney	2008	Southeast	[56]
*Morbillivirus*	*Myotis riparius*/*Chiroptera*	Insectivorous	Mix	2013	North	[54]
*Morbillivirus*	*Phyllostomus hastatus*/Phyllostomidae	Nectarivore	Mix	2013–2014	Southeast	[54]
*Jeilongvirus*	*Carollia perspicillata*/Phyllostomidae	Insectivorous	Mix	2013–2014	Southeast	[54]
*Jeilongvirus*	*Diaemus youngi*/Phyllostomidae	Hematophagous	Mix	2013	Southeast	[54]
*Jeilongvirus*-like	*Diaemus youngi*/Phyllostomidae	Hematophagous	Mix	2013	Southeast	[55]

^a^ Putatively named *Macrojêvirus* [61]; Mix: urine and/or oral swab and/or tissue.

## Data Availability

Not applicable.
